# Supplementary material for: Novel N-normetazocine Derivatives with Opioid Agonist/Sigma-1 Receptor Antagonist Profile as Potential Analgesics in Inflammatory Pain
Source: Molecules. 2022 Aug 12;27(16):5135. doi: 10.3390/molecules27165135 (PMC9413390; doi:10.3390/molecules27165135)
Supplement: Supplementary file 1 [file molecules-27-05135-s001.zip › molecules-1839129-supplementary.pdf]

Supporting Information

Novel *N*-normetazocine Derivatives with Opioid Agonist /Sigma-1 Receptor Antagonist Profile as Potential Analgesics in Inflammatory Pain

Rita Turnaturi[1], Santina Chiechio[2,3], Lorella Pasquinucci[1*], Salvatore Spoto[2], Giuliana Costanzo[4], Maria Dichiara[1], Silvia Piana[1], Margherita Grasso[3], Emanuele Amata[1], Agostino Marrazzo[1], Carmela Parenti[2]

1 Department of Drug and Health Sciences, Medicinal Chemistry Section, University of Catania, Viale A. Doria 6, 95125 Catania, Italy
2 Department of Drug and Health Sciences, Section of Pharmacology and Toxicology, University of Catania, Viale A. Doria 6, 95125 Catania, Italy
3 Oasi Research Institute—IRCCS, 94018 Troina, Italy
4 Department of Biomedical and Biotechnological Sciences, University of Catania, Via Santa Sofia, 97, 95123 Catania, Italy
* Correspondence: lpasquin@unict.it; Tel.: +39-095-738-4273

Table of contents

Tanimoto structural similarity index (Tsc) – Tables S1 and S2 S2

General procedure for the synthesis of tosylate intermediates (**4**-**6**) S2-S3

NMR spectra of compounds **7**-**9**. (Figure S1-S6) S4-S6

Table S3. Elemental analysis data for compounds **7**-**9** S7

Synthesis of PRE-84 S7

NMR spectra of PRE-84 (Figure S7) S8

Tanimoto structural similarity index (Tsc)

We have evaluated the structural similarity between our LP compounds and standards used in competition binding assay calculating the Tanimoto coefficient (Tc). The Tc yields values within the range 0 to 1 [44]. The distribution of Tc values over this range depends on the fingerprint that is used and the compound characteristics. The data are obtained using the online software https://chemminetools.ucr.edu/similarity/. A comparison between compound **1** and opioid receptor standards, and compound **7** and sigma receptors standards has been performed. Data obtained, reported in the tables 1 and 2, are confirmed by the results of the binding assays.

Table S1: Tc values *versus* opioid standards.

**(−)-2*R*/*S*-LP2 (1)**	**AP Tanimoto**	**MCS Tanimoto**	**MCS Size**	**MCS Min**	**MCS Max**
DAMGO	0.177549	0.2800	14	0.5385	0.3684
U69,593	0.221805	0.4054	15	0.5769	0.5769
Naltrindole	0.307325	0.4872	19	0.7308	0.5938

Table S2: Tc values *versus* sigma standards.

(+)-2*R*/*S*-LP2 (**7**)	**AP Tanimoto**	**MCS Tanimoto**	**MCS Size**	**MCS Min**	**MCS Max**
ALOPERIDOLO	0.186131	0.4054	15	0.5769	0.5769
PENTAZOCINA	0.455497	0.6552	19	0.8636	0.7308
DTG	0.135392	0.1892	7	0.3889	0.2692
BD-1063	0.17602	0.4333	13	0.7647	0.5000

[44] Vogt M, Bajorath J. Modeling Tanimoto Similarity Value Distributions and Predicting Search Results. Mol Inform. 2017; 36(7). doi: 10.1002/minf.201600131.

General procedure for the synthesis of tosylate intermediates (4-6)

To a solution of 2-methoxy-2-phenylethanol or (*R*)-(−)-2-methoxy-2-phenylethanol or (*S*)-(+)-2-methoxy-2-phenylethanol (3.29 mmol, 1 eq) in dry CH_2Cl_2 (30 mL) Bu_2SnO was added (0.066 mmol, 0.02 eq) followed by the addition of pTsCl (3.29 mmol, 1 eq) and TEA (3.29 mmol, 1 eq). The reaction mixture was stirred at rt for 3h. The reaction mixture was quenched by adding water. The solution was extracted with CH_2Cl_2. The organic layer was washed with a saturated aqueous solution of brine and dried over anhydrous Na_2SO_4. The solvent was evaporated to provide a solid which was purified by column chromatography (silica gel, AcOEt : C_6H_{12} 3:7) to yield the desired the compounds (**4**-**6**).

*(2R/S)-2-methoxy-2-phenylethyl 4-methylbenzenesulfonate (**4**).* Colorless oil (70%). IR ($CHCl_3$) 3020, 1598, 1451, 1358, 1176 cm^{-1}. ^1H NMR (200 MHz, $CHCl_3$) δ 7.76-7.72 (m, 2H), 7.32-7.26 (m, 7H), 4.42-4.38 (m, 1H), 4.09-4.03 (m, 2H), 3.23 (s, 3H), 2.44 (s, 3H).

*(R)-2-methoxy-2-phenylethyl 4-methylbenzenesulfonate (**5**).* Waxy solid (94%). Mp: 52-55 °C. $[\alpha]_D^{25}$ = − 42.9° (c 1.05, $CHCl_3$). IR ($CHCl_3$) 3019, 2943, 1598, 1456, 1362, 1215, 1176 cm^{-1}. ^1H NMR (200 MHz, $CDCl_3$): δ 7.76-7.72 (m,

2H), 7.32-7.26 (m, 7H), 4.42-4.39 (m, 1H), 4.09-4.03 (m, 2H), 3.23 (t, 3H), 2.44 (s, 3H). ^{13}C NMR (50 MHz, $CDCl_3$) δ 144.79, 136.63, 132.91, 129.69, 128.62, 128.55, 127.83, 126.85, 81.15, 72.79, 56.98, 21.46.

*(S)-2-methoxy-2-phenylethyl 4-methylbenzenesulfonate (**6**).* Waxy solid (74%). Mp 54-55 °C. $[\alpha]_D^{25}$ = + 42.2° (c 1.02, $CHCl_3$). IR ($CHCl_3$) 3019, 2934, 1598, 1455, 1361, 1215, 1176 cm^{-1}. ^{1}H NMR (200 MHz, $CHCl_3$) δ 7.76-7.72 (m, 2H), 7.43-7.22 (m, 7H), 4.44-4.39 (m, 1H) 4.09-4.03 (m, 2H), 3.22 (t, 3H), 2.44 (s, 3H). ^{13}C NMR (50 MHz, $CHCl_3$) δ 144.67, 136.73, 132.92, 129.72, 128.65, 128.57, 127.86, 126.88, 81.12, 72.85, 57.01, 21.59.

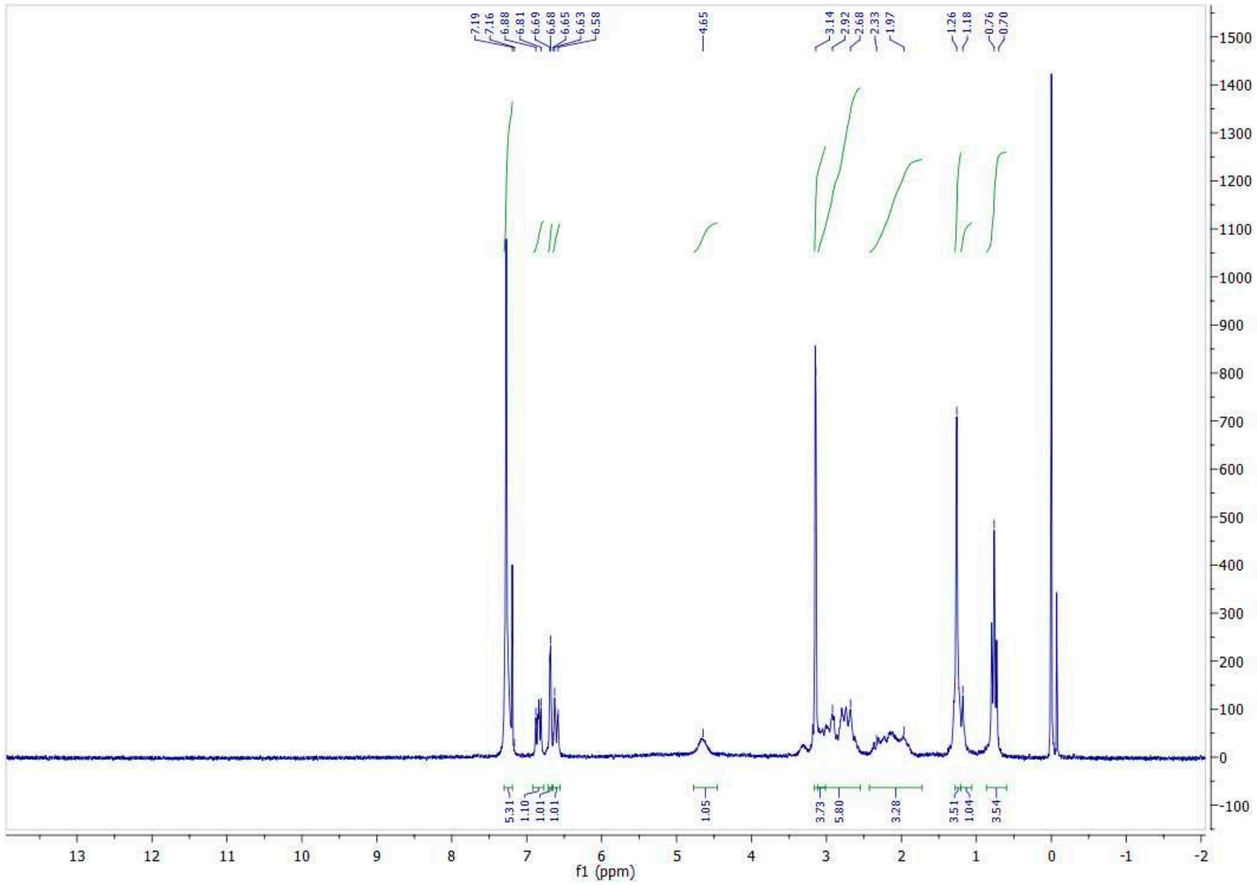

Figure S1. ^{1}H-NMR (200 MHz, $CDCl_3$, free base) spectra of compound **7**.

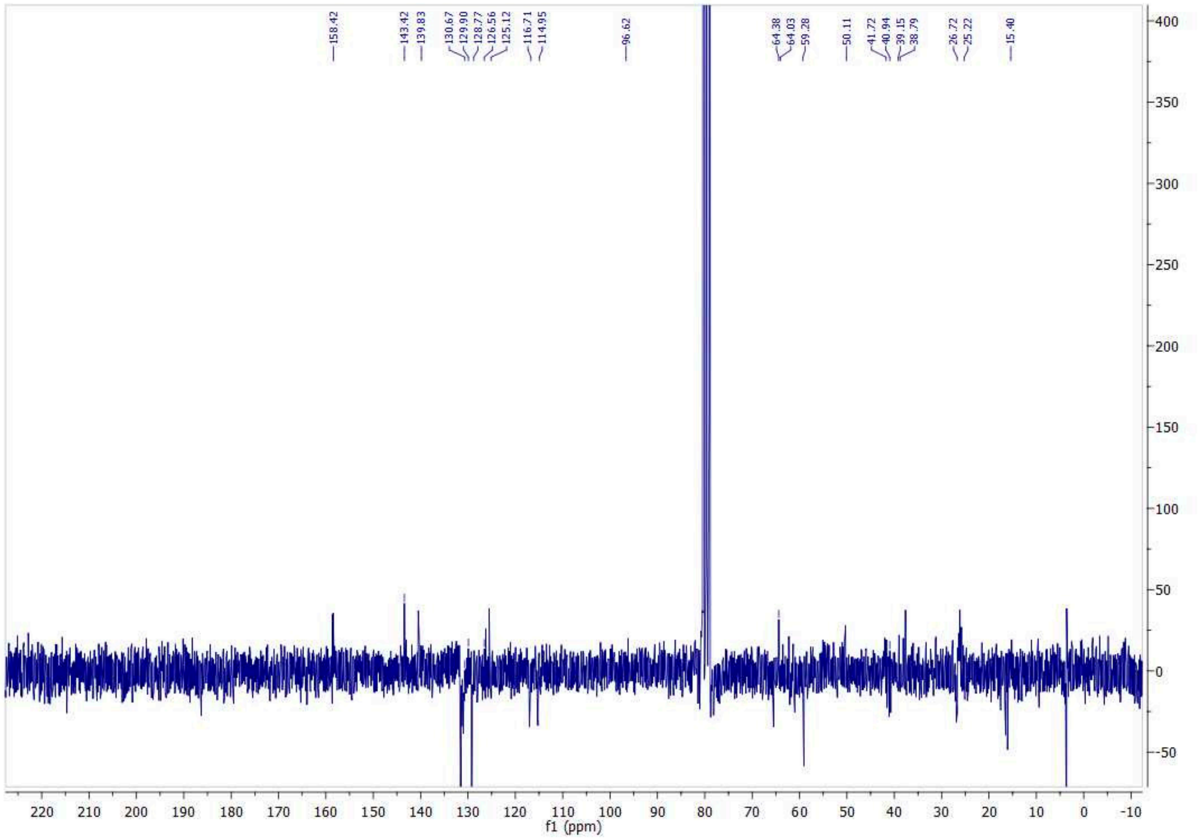

Figure S2. ^{13}C-NMR (50 MHz, $CDCl_3$, free base) spectra of compound **7**.

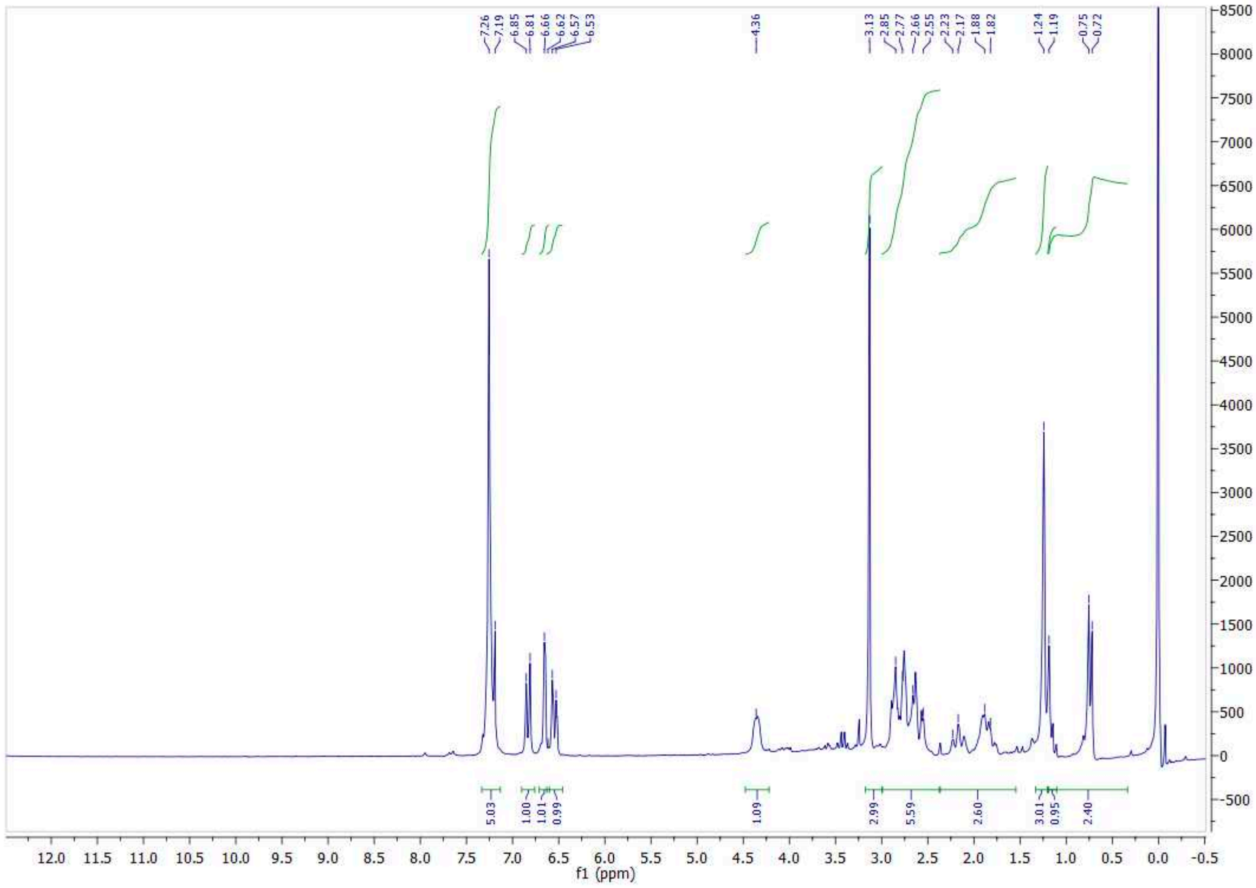

Figure S3. ^{1}H-NMR (200 MHz, $CDCl_3$, free base) spectra of compound **8**.

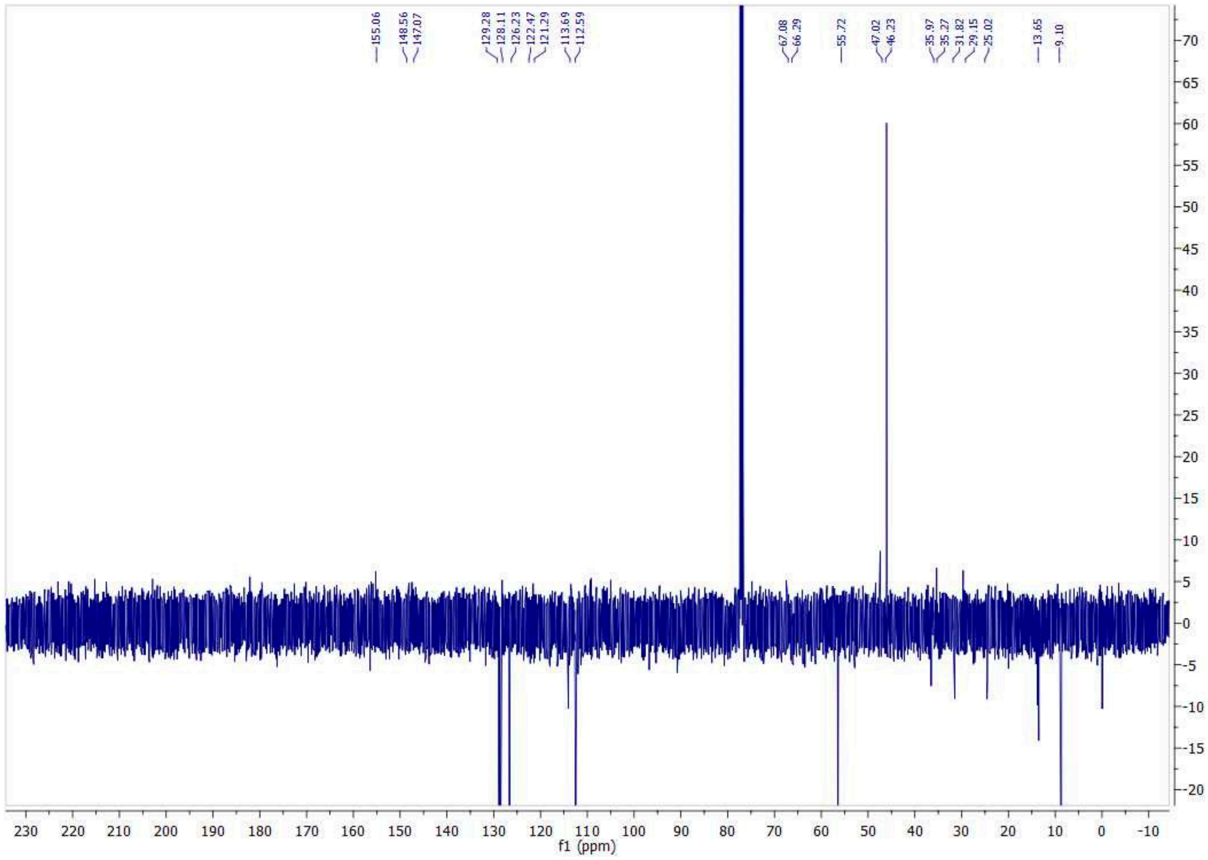

Figure S4. ^{13}C-NMR (50 MHz, $CDCl_3$, free base) spectra of compound **8**.

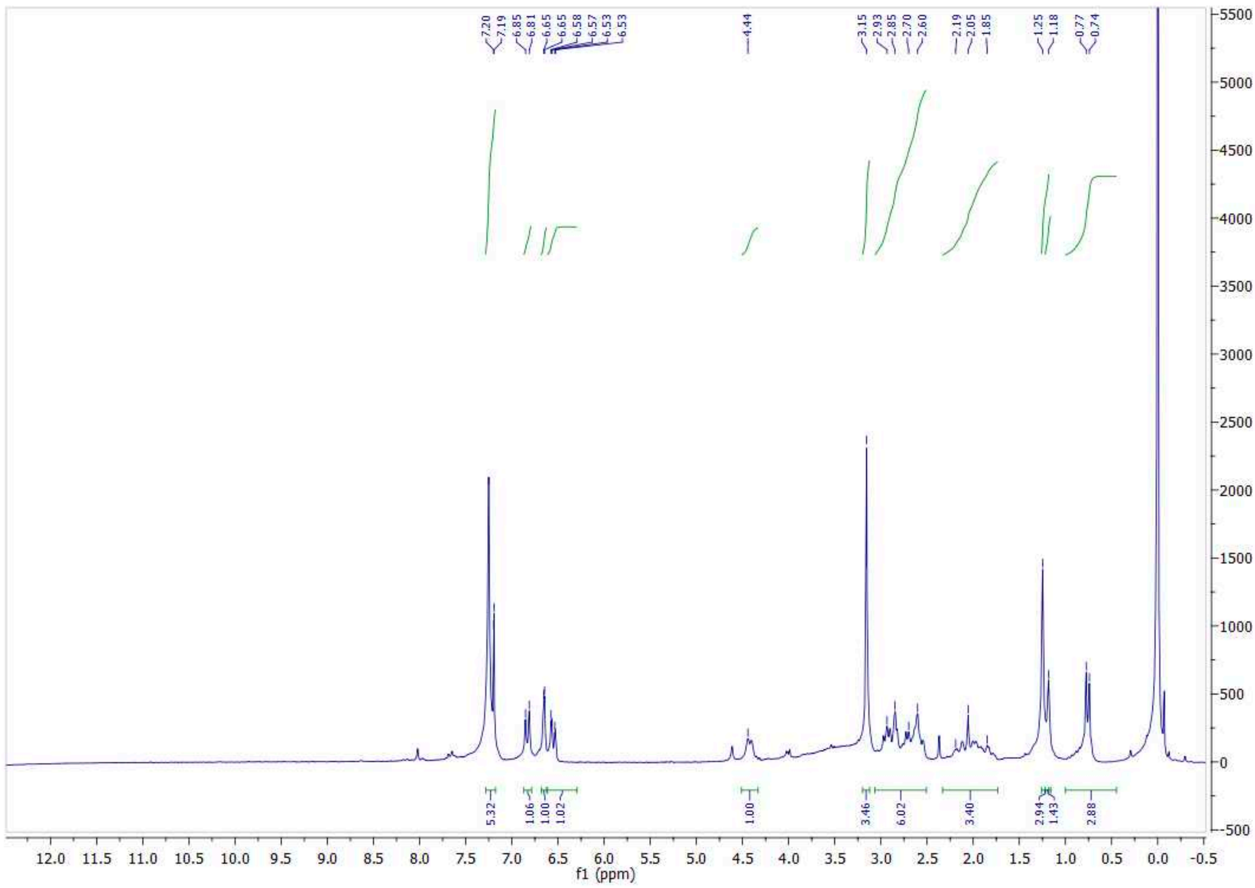

Figure S5. ^{1}H-NMR (200 MHz, $CDCl_3$, free base) spectra of compound **9**.

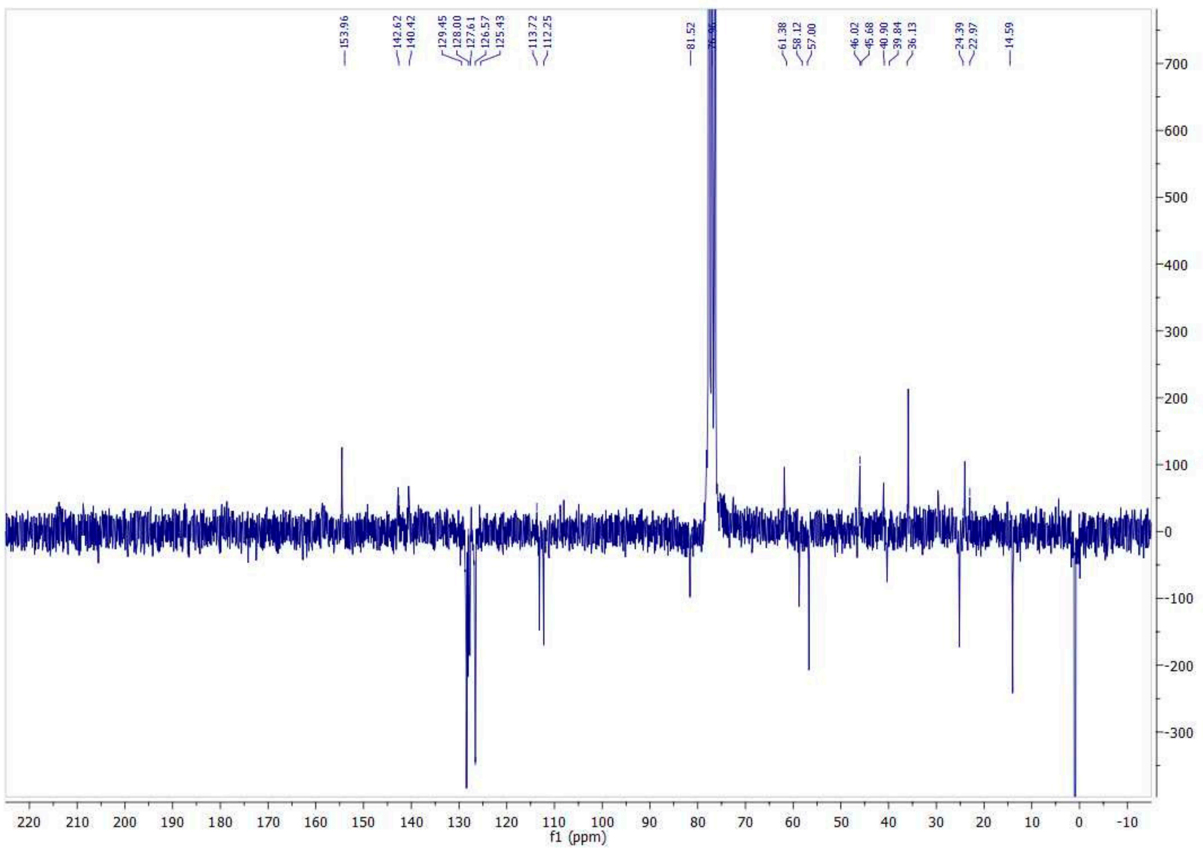

Figure S6. ^{13}C-NMR (50 MHz, $CDCl_3$, free base) spectra of compound **9**.

Table S3. Elemental analysis data for compounds **7-9**.

			Calcd			Found		
**Compd**	**Formula**	**Mw**	**C**	**H**	**N**	**C**	**H**	**N**
**7**	$C_{23}H_{30}ClNO_2$	387.94	71.21	7.79	3.61	71.35	7.85	3.41
**8**	$C_{23}H_{30}ClNO_2$	387.94	71.21	7.79	3.61	71.41	7.98	3.38
**9**	$C_{23}H_{30}ClNO_2$	387.94	71.21	7.79	3.61	71.40	7.90	3.40

Synthesis of PRE-84

2-morpholinoethyl 1-phenylcyclohexane-1-carboxylate.

1-Phenyl-1-cyclohexanecarboxylic acid (4.895 mmoli, 1 eq) was dissolved in $SOCl_2$ (14.686 mmoli, 3 eq) and heated (80 °C) for 4 hours. Excess $SOCl_2$ was removed under vacuum. The resulting oil was dissolved in methylene chloride (5 ml) containing triethylamine (5.384 mmoli, 1.1 eq), and 4-(2-hydroxyethyl) morpholine (7.342 mmoli, 1.5 eq) was added. The mixture was stirred overnight at room temperature. After the solution was diluted with methylene chloride and washed with aqueous $NaHCO_3$, the solvent was removed under vacuum. The obtained orange oil was purified with flash-chromatography using as gradient eluent C_6H_{12}/EtOAc 90-10 70-30 (v/v). The final compound was converted to the hydrochloride salt with HCl solution **(**2.0 M in diethyl ether).

White solid (30%) ^{1}H NMR (200 MHz, $CDCl_3$, free base) δ 7.42-7.21 (m,5H); 4.29 (t, 2H); 3.58 (t, 4H); 2.51 (t, 4H); 2.32 (t, 4H); 1.71-1.54 (m, 8H).

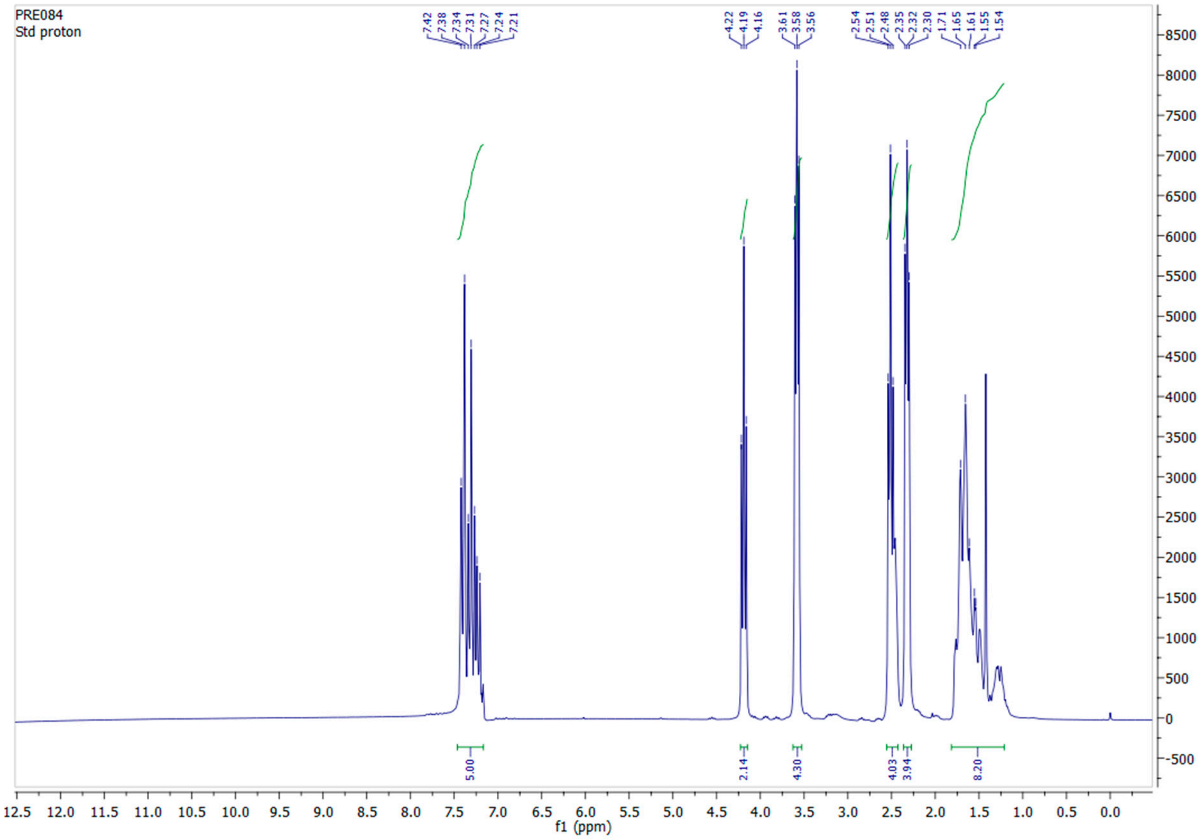

Figure S7. ^{1}H-NMR (200 MHz, CDCl3, free base) spectra of **PRE-084**.
